# Neurodegeneration in MS and NMO: The Eye and the Blood

**DOI:** 10.1155/2011/842797

**Published:** 2011-11-30

**Authors:** Axel Petzold, Jeroen J. G. Geurts, Ichiro Nakashima, Helmut Butzkueven, Robert Weissert

**Affiliations:** ^1^UCL Institute of Neurology, Queen Square, London WC1N 3BG, UK; ^2^VU University Amsterdam, 1081 HV Amsterdam, The Netherlands; ^3^Tohoku University, Aoba-ku 980-8576, Japan; ^4^Department of Medicine, University of Melbourne, Parkville 3010, Australia; ^5^Department of Neurology, University of Regensburg, Universitaetsstrasse 84, 93053 Regensburg, Germany

Not surprisingly, patients perceive a considerable reduction of quality of life following impairment of their visual function. What perhaps few of us in the field of multiple sclerosis realized is that patients rank loss of visual function second only to loss of mobility [1]. Fittingly, this first of a series of special issues comprises three reviews on the visual system in multiple sclerosis and related disorders. From Canada, we have F. Costello telling us that the saying “the patient does not see anything, nor does the doctor” may become history with the broader application of a rapidly evolving technology, optical coherence tomography (OCT) in “*Evaluating the use of optical coherence tomography in optic neuritis.*” The data discussed in this review provides compelling evidence that loss of retinal axons can be quantified with high accuracy. Further extending this new technology, S. Noval et al., from Spain, conclude that OCT may “represent an objective outcome measure” for treatment trials in “*Optical coherence tomography in multiple sclerosis and neuromyelitis optica*: an update” Indeed, several therapeutic trials in MS utilizing OCT as a secondary outcome measure are in advanced planning stages. Given the ease and speed of the examination, OCT could become a valuable tool for the MS community. Given the anatomical constraints of the human nervous system, the technique is unlikely to pose a challenge to firmly established magnetic resonance imaging (MRI) of the brain. So those of us who spend hours sleeping as control subjects in ever-increasing magnetic fields may continue collecting colored ear plugs. In fact, the comprehensive review on MRI of the visual pathways by C. Pfueller and F. Paul (Germany) tells why these two methods are complementary (in “*Imaging the visual pathway in neuromyelitis optica*”). Nonetheless, assessment of retinal nerve fibre layer thickness by OCT is a readily accessible measure, unlike cerebral volume, and one could imagine using this quantitative marker for routine monitoring of MS progression in the clinic in the near future.

The focus on the elusive cause of MS has shifted in this special issue to possible mechanisms driving neurodegeneration, the major cause for sustained disability. From Italy we have B. Tavazzi et al. presenting biomarker data which suggests that oxidative damage hangs as a Damocles sword over the impaired energy metabolism in axons damaged in multiple sclerosis in “*Serum metabolic profile in multiple sclerosis patients.*” One perennially popular candidate out of Pandora's box of oxidative damage is iron. In a beautiful translational review M. Khalil et al. from Austria summarize the evidence linking iron to neurodegeneration in MS (in “*Iron and neurodegeneration in multiple sclerosis*”). Neurodegeneration in MS is a process which can be captured in time by using specific biomarkers as illustrated by two comprehensive reviews by I. Dujmovic (Serbia) and M. M. Gresle et al. (Australia) (“*Cerebrospinal fluid and blood biomarkers of neuroaxonal damage in multiple sclerosis*” and “*Neurofilament proteins as body fluid biomarkers of neurodegeneration in multiple sclerosis*”). The lecture of these reviews brings to the readers mind why it is so important to have biomarker research standardized, reflected in a restated and updated consensus paper on this issue by C. Teunissen et al. from The Netherlands (titled “*Consensus guidelines for CSF and blood biobanking for CNS biomarker studies*”). Would it not be nice if we could measure neurodegeneration from a patient's blood sample similar to what cardiologists can do with troponin? M. J. Eikelenboom et al. also from The Netherlands remind us that our experience with cerebrospinal fluid data (in “*Cerebrospinal fluid and blood biomarkers of neuroaxonal damage in multiple sclerosis*” and “*Neurofilament proteins as body fluid biomarkers of neurodegeneration in multiple sclerosis*”) cannot readily be extrapolated to the blood during the chronic phase of the disease in “*Blood and CSF biomarker dynamics in multiple sclerosis: implications for data interpretation.*” Unfortunately during the progressive phase of multiple sclerosis, there is no general convincing evidence for the efficacy of disease-modifying drugs, and experimental options such as repeated intrathecal steroids are still under investigation as M. Abu-Mugheisib et al. from Germany summarize (in “*Repeated intrathecal triamcinolone acetonide administration in progressive multiple sclerosis*: a review”). In this context the anecdotal observation by G. T. Plant et al. from London that steroid treatment in the hyperacute phase of optic neuritis prevents loss of vision is remarkable (in “*Hyperacute corticosteroid treatment of optic neuritis at the onset of pain may prevent visual loss: a case series*”). The eight patients reported in this paper appear as a David compared to the large Goliath-like dataset of the Optic Neuritis Treatment Trial (ONTT) which demonstrated that steroids did not change the outcome of visual function. Nevertheless, small patient numbers might be sufficient if the disease biology is clear-cut: James Lind only investigated twelve sailors to find the cause and treatment for scurvy [2]. The careful examination of patients with acute optic neuritis by F. Costello et al. using OCT in “*Exploring the association between retinal nerve fiber layer thickness and initial magnetic resonance imaging findings in patients with acute optic neuritis*” may just provide the outcome measure needed for a future treatment trial in this direction. 
